# Characterizing electron-collecting CdTe for use in a 77 ns burst-rate imager

**DOI:** 10.1107/S160057752400643X

**Published:** 2024-08-07

**Authors:** Lena A. Franklin, Nicholas J. Brown, Sol M. Gruner, Elida Met-Hoxha, Mark W. Tate, Julia Thom-Levy

**Affiliations:** a Physics Department Cornell University,Ithaca NY USA; b CHESS Cornell University,Ithaca NY USA; University of Tokyo, Japan

**Keywords:** pixel array detector, cadmium telluride, X-ray detector, high-*Z* sensor

## Abstract

The Keck-PAD (pixel array detector) was developed at Cornell as a burst-rate imager capable of recording images from successive electron bunches (153 ns period) from the Advanced Photon Source (APS). Both Si and hole-collecting Schottky CdTe have been successfully bonded to this ASIC (application-specific integrated circuit) and used with this frame rate. The facility upgrades at the APS will lower the bunch period to 77 ns, which will require modifications to the Keck-PAD electronics to image properly at this reduced period. In addition, operation at high X-ray energies will require a different sensor material having a shorter charge collection time. For the target energy of 40 keV for this project, simulations have shown that electron-collecting CdTe should allow >90% charge collection within 35 ns. This collection time will be sufficient to sample the signal from one frame and prepare for the next. 750 µm-thick electron-collecting Schottky CdTe has been obtained from Acrorad and bonded to two different charge-integrating ASICs developed at Cornell, the Keck-PAD and the CU-APS-PAD. Carrier mobility has been investigated using the detector response to single X-ray bunches at the Cornell High Energy Synchrotron Source and to a pulsed optical laser. The tests indicate that the collection time will meet the requirements for 77 ns imaging.

## Abstract 2

The characterization of electron-collecting CdTe for use in a 77 ns burst-rate charge-integrating detector is described.

## 1. Introduction

Pixel array detectors (PADs) have been an important technology in X-ray science for several decades. The two main components of a PAD are a sensor layer, which absorbs incident radiation and produces a signal proportional to the radiation intensity, and an application-specific integrated circuit (ASIC) which performs signal processing. Silicon is a commonly used material in the sensor layer because of its quality, cost and availability. However, the low atomic number of silicon means that it offers poor stopping power for high-energy X-rays. Due to silicon’s low quantum efficiency at high energies, higher atomic number (high-*Z*) materials are used for detectors designed to operate at energies above 20 keV. One of the first high-*Z* materials to be implemented for radiation detection was cadmium telluride (CdTe), which offers considerably greater quantum efficiency compared with silicon at higher energies. However, the charge collection time for CdTe is considerably longer than for silicon, with the mobility of the holes being the most limiting factor for high-speed imaging (Ariño-Estrada *et al.*, 2014 ▸). The collection time could be improved in CdTe by opting for thinner sensors, however commercially available sensors are only offered with relatively large thicknesses (greater than ∼750 µm). Thinner wafers of CdTe would require the development of new procedures for processing and handling.

The Keck-PAD was developed at Cornell to perform burst-rate imaging and was designed to match the 153 ns bunch spacing at the Advanced Photon Source (APS) (Philipp *et al.*, 2016*a* ▸). Silicon and hole-collecting Schottky CdTe sensors with a 150 µm pixel pitch have been successfully used at the APS with this bunch pattern and at the Cornell High Energy Synchrotron Source (CHESS), using X-rays spanning a wide range of energies (Lambert *et al.*, 2014 ▸; Becker *et al.*, 2017 ▸). Upgrades to the APS are underway which will decrease bunch spacing to 77 ns. Operation at this bunch spacing will require some modification to the signal-processing ASIC layer, which will be discussed in a future publication.

The charge collection time from a silicon sensor is fast enough to be compatible with this frame rate. This is not the case for 750 µm-thick CdTe hole-collecting material (Becker *et al.*, 2017 ▸), necessitating an alternative to be found for operation at higher X-ray energies. For certain X-ray energies, including the target X-ray energy of 40 keV for which this detector is being developed, an interesting solution arises by using a 750 µm-thick electron-collecting CdTe sensor.

**Figure 1:**
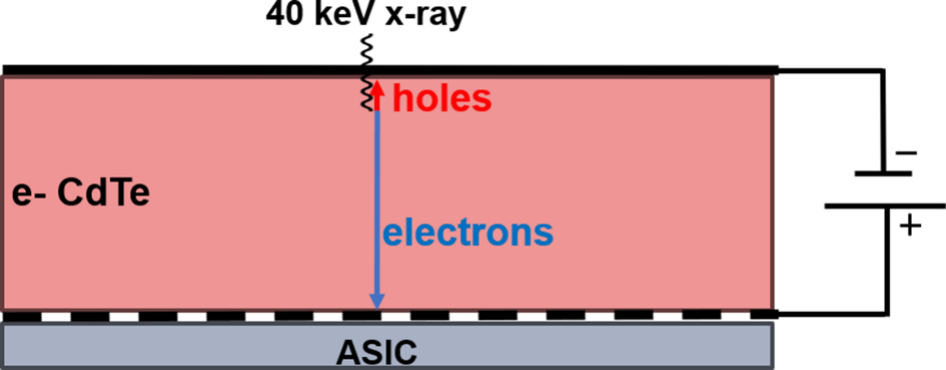
Electron-collecting sensor cross section (750 µm thick). The majority of 40 keV photons will be absorbed in the top 90 µm of the sensor. The holes created by the photon absorption travel the relatively short distance to the top surface and the electrons travel the greater distance to the pixelated readout on the ASIC side. Note that, for hole-collecting material, the hole and electrons will each travel in the opposite direction.

**Figure 2:**
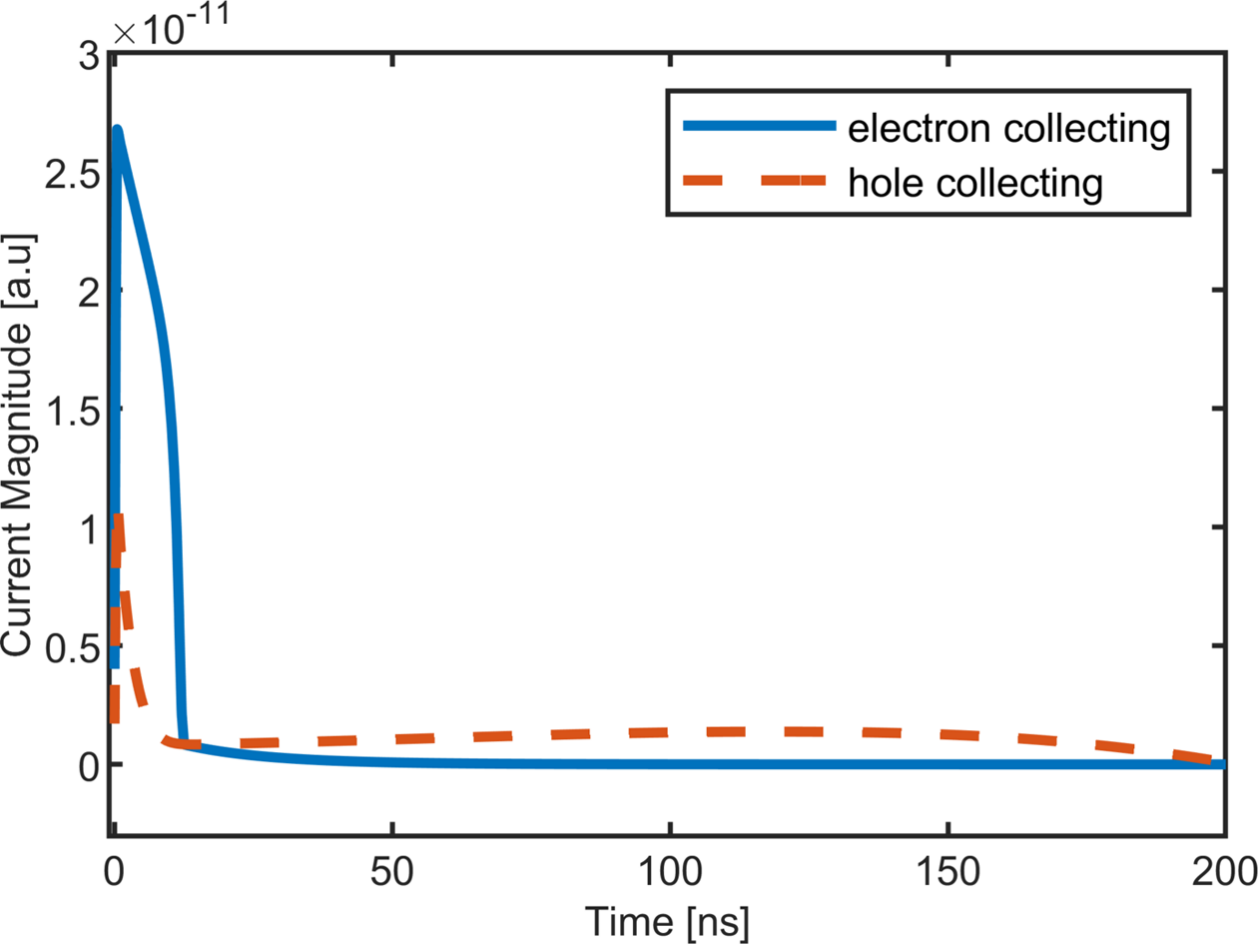
Simulation of the current induced by an ensemble of 40 keV photons in 750 µm-thick hole-collecting (dashed red line) and electron-collecting (solid blue line) CdTe sensors. The simulations were done using a charge collection area much wider than the sensor thickness. The experimental response shown in Figs. 6 ▸–8 ▸ ▸ was also computed by integrating the incident signal over a wide region of interest.

At an energy of 40 keV and below, most of the photons will be absorbed in the top 90 µm of the sensor, creating a cloud of electron–hole pairs. The electrons and holes drift towards the opposite faces of the sensor, with the electrons drifting about ten times faster than the holes. In the case of hole-collecting material, the faster electrons drift ∼90 µm to the entrance side, while the slower holes must traverse ∼660 µm to the ASIC-bonded side. For electron-collecting material, the holes will now travel a comparatively short distance as compared with the faster electrons, as shown in Fig. 1 ▸, reducing the overall collection time considerably. Fig. 2 ▸ shows a simulation (Becker, 2010 ▸) of the current induced by a 40 keV photon in a hole compared with electron-collecting CdTe at ±400 V bias, with the current persisting for much longer in the hole-collecting case. The time to collect 95% of the charge drops from 183 ns to 15 ns.

In this study, we characterize the properties of 750 µm-thick electron-collecting Schottky CdTe bonded to two different ASIC readout platforms, the Keck-PAD, operating as a burst-mode imager, as well as the CU-APS-PAD system (Gadkari *et al.*, 2022 ▸), which offers a high dynamic range and a lower inherent noise floor. Charge collection speeds were investigated using illumination from a pulsed optical laser and the single bunch response was monitored at the Cornell High Energy Synchrotron Source (CHESS).

## 2. Detector systems and material properties

The Keck-PAD (Philipp *et al.*, 2016*b* ▸) and the CU-APS-PAD ‘MM-PAD-2.1’ (Gadkari *et al.*, 2022 ▸) are charge-integrating ASICs developed by Cornell and have previously been used with hole-collecting CdTe sensor layers (Becker *et al.*, 2016 ▸). Both ASICs feature an array of 128 × 128 pixels with a pixel area of (150 µm)^2^.

The Keck-PAD is designed to operate as a burst-rate imager, taking a burst of eight images at up to 10 MHz. After burst imaging is complete, images are read out at a slower rate of around one image per millisecond. The pixel values are digitized in the readout electronics to give an analog digitized unit (ADU). In the highest gain setting used in this study, the Keck-PAD has an equivalent read noise of 5.6 keV with a silicon sensor. Data taken on the Keck-PAD were used to assess the charge collection speed and to study the effects of polarization within the material.

The CU-APS-PAD images continuously at up to 10 kHz and incorporates two-stage adaptive gain allowing for a high dynamic range and imaging at fluxes as high as 10^10^ photons pixel^−1^ s^−1^ (Gadkari *et al.*, 2022 ▸). The equivalent read noise for this platform is 2.5 keV for silicon and 3.72 keV for CdTe (Gadkari *et al.*, 2022 ▸). The CU-APS-PAD was used to take photon pinhole spectra.

**Figure 3:**
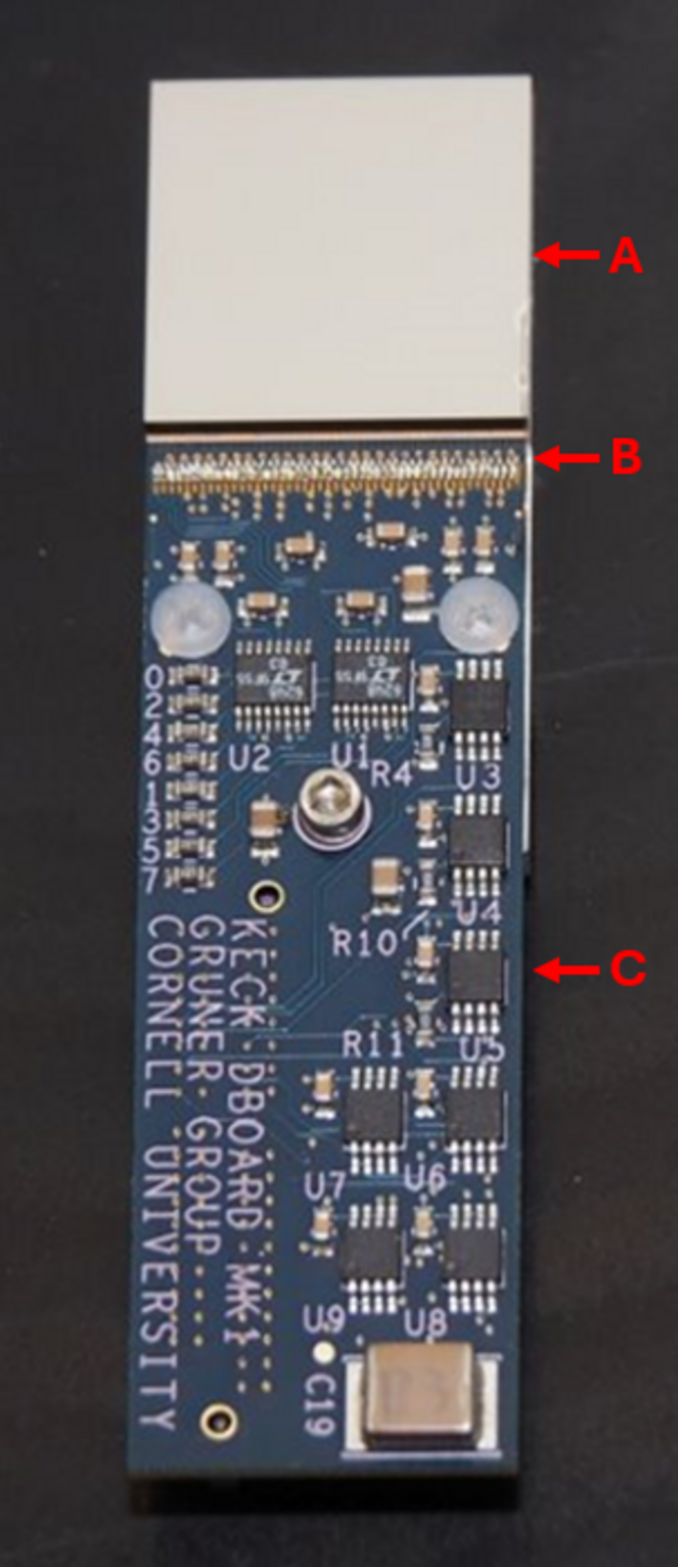
Sensor module formed from CdTe sensor layer bonded to a Keck-PAD ASIC. The sensor layer (A) is bump-bonded to an underlying ASIC (not visible) which is wire-bonded (B) to the printed circuit board (C).

The sensor material used in this study is 750 µm-thick Schottky CdTe produced by Acrorad Co. (Okinawa, Japan). The CdTe is bonded pixel by pixel to the ASIC to produce a sensor module as shown in Fig. 3 ▸. For the Keck-PAD, bonding using gold stud bumps applied to the ASIC layer coupled with a stenciled, conductive epoxy applied to the sensor was performed by Polymer Assembly Technology (6581 Belding Rd NE #102, Rockford, MI, USA). For the CU-APS-PAD module, bonding was performed by Oy Direct Conversion Ltd (Finland). The sensor modules for both systems are placed in evacuated housings with a transparent vacuum window. The temperature of the sensor is controlled to within ±0.1°C using a thermoelectric cooler.

## 3. Charge transport measurements

Carrier mobility and charge collection times can be studied using pulsed optical and X-ray sources in conjunction with the Keck-PAD system. By varying the wavelength of the incident photon source (optical or X-ray), one can change the depth profile of the generated electron/hole pairs. Measuring the response at different depth profiles as a function of bias voltage allows the electron and hole mobility to be separated and measured.

**Figure 4:**
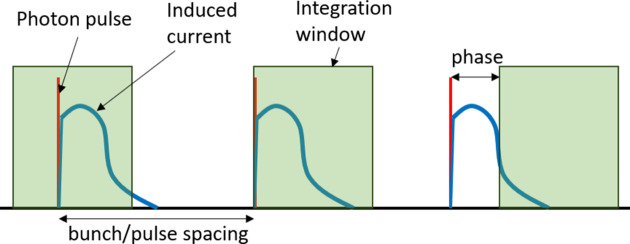
The procedure for measuring sensor current response with an integrating detector. This figure shows three successive photon pulses (red) and the corresponding induced current pulses collected at the pixel input (blue). The induced current is collected only during the times indicated by the green charge integration window. By phasing the start of the integration period with respect to the arrival of the photon pulse, one can map the entire induced current response.

**Figure 5:**
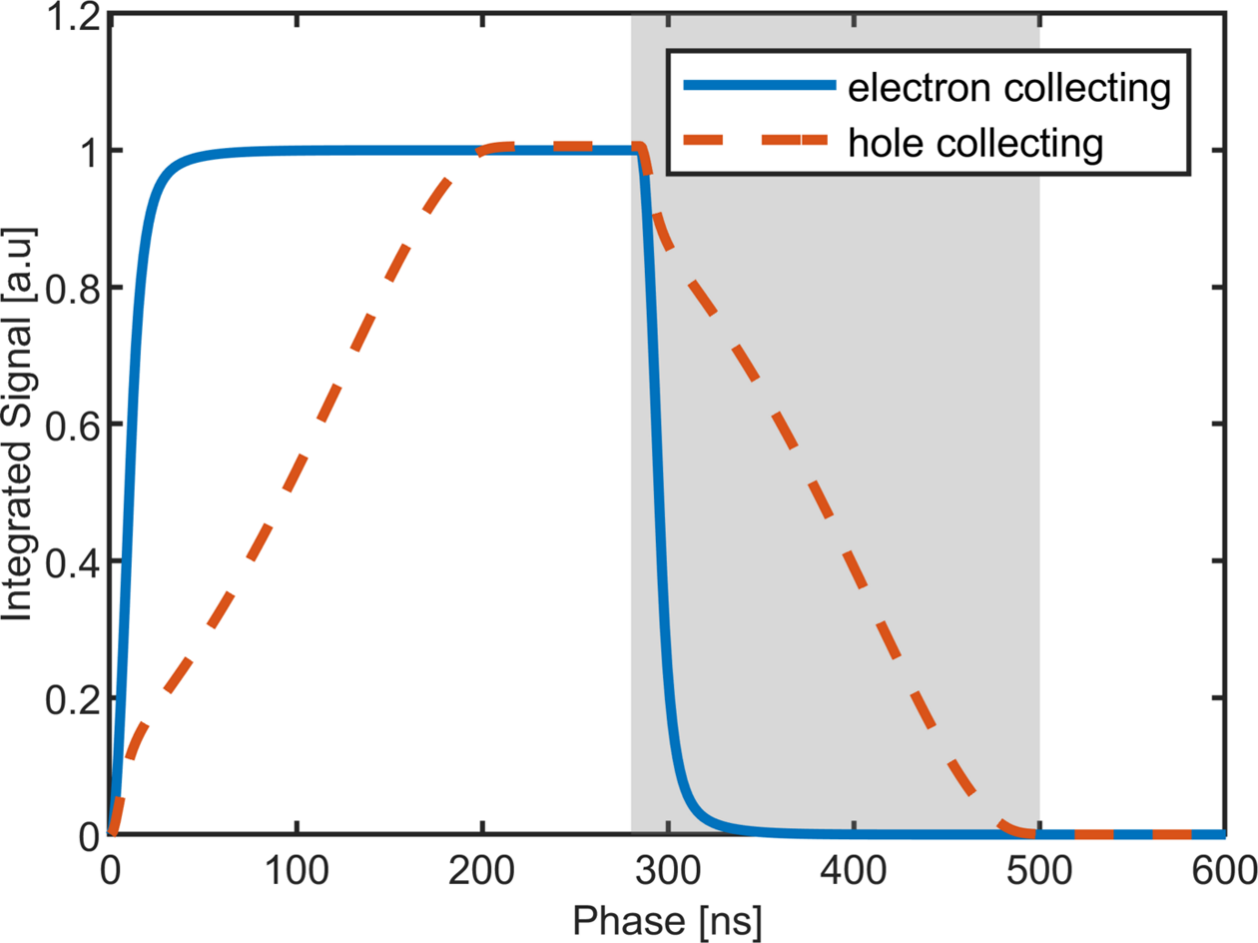
Signal (integrated current) measured for the current pulses shown in Fig. 2 ▸ as a function of the phase delay between the induced current pulse and a 300 ns-long charge integration window. The length of the window is chosen to be longer than the length of the current pulse. The curves are normalized to the total charge contained in the pulse. These curves directly show the total fraction of charge that can be collected within a given time window. The rising and falling edges contain equivalent information about the charge collection dynamics. We have chosen to show the falling edge (as highlighted by the gray box) in Figs. 6 ▸ and 7 ▸ since the ASIC amplifier is known to be faster on the falling edge.

The Keck-PAD measures the total integrated charge accumulated over a defined window in time rather than the instantaneous induced current during the exposure. However, by moving the integration window with respect to the arrival of the photon pulse, one can map out the shape of the current pulse (Fig. 4 ▸). As one advances the phase of the integration window, there is a period where the signal rises over the charge collection time, a constant period over which the pulse is entirely within the integration window, and a period where the signal falls, as shown in Fig. 5 ▸. The width of the integration window is chosen to be greater than the duration of the current pulse but shorter than the pulse repetition time. For an ideal amplifier in the pixel, the rising and falling periods should produce symmetric curves. For the Keck-PAD system, however, the amplifier response was seen to be much faster on the falling edge, suggesting that the amplifier was slew rate limited during the rising period. Measurements using different sensor biases showed the amplifier slew time was limited to around 20 ns on the falling edge.

To determine charge carrier mobilities, the normalized intensity (proportional to the integral of the induced current) per pixel is plotted against the relative phase between the photon pulse and the beginning of the integration period. The results are compared with simulations from a custom MATLAB code (Becker, 2010 ▸), modified to account for the effects of the 5.2 ns RC time constant of the amplifier of the Keck-PAD. The code determines the electron–hole creation profile of a given photon source, determines the induced current using the Shockley–Ramo theorem, and gives the results of integrating such an induced current given the delays present in the detector electronics. The rise and fall time of the signal amplitudes as a function of the phase between the beginning of integration and the photon pulse is used to measure the charge collection time of the material. A limiting factor is amplifier slew rate, which can artificially delay the initial rapid drop-off of the falling edge for bias voltage magnitudes above 300 V.

### 3.1. Electron and hole mobility characterized using a pulsed laser

**Figure 6:**
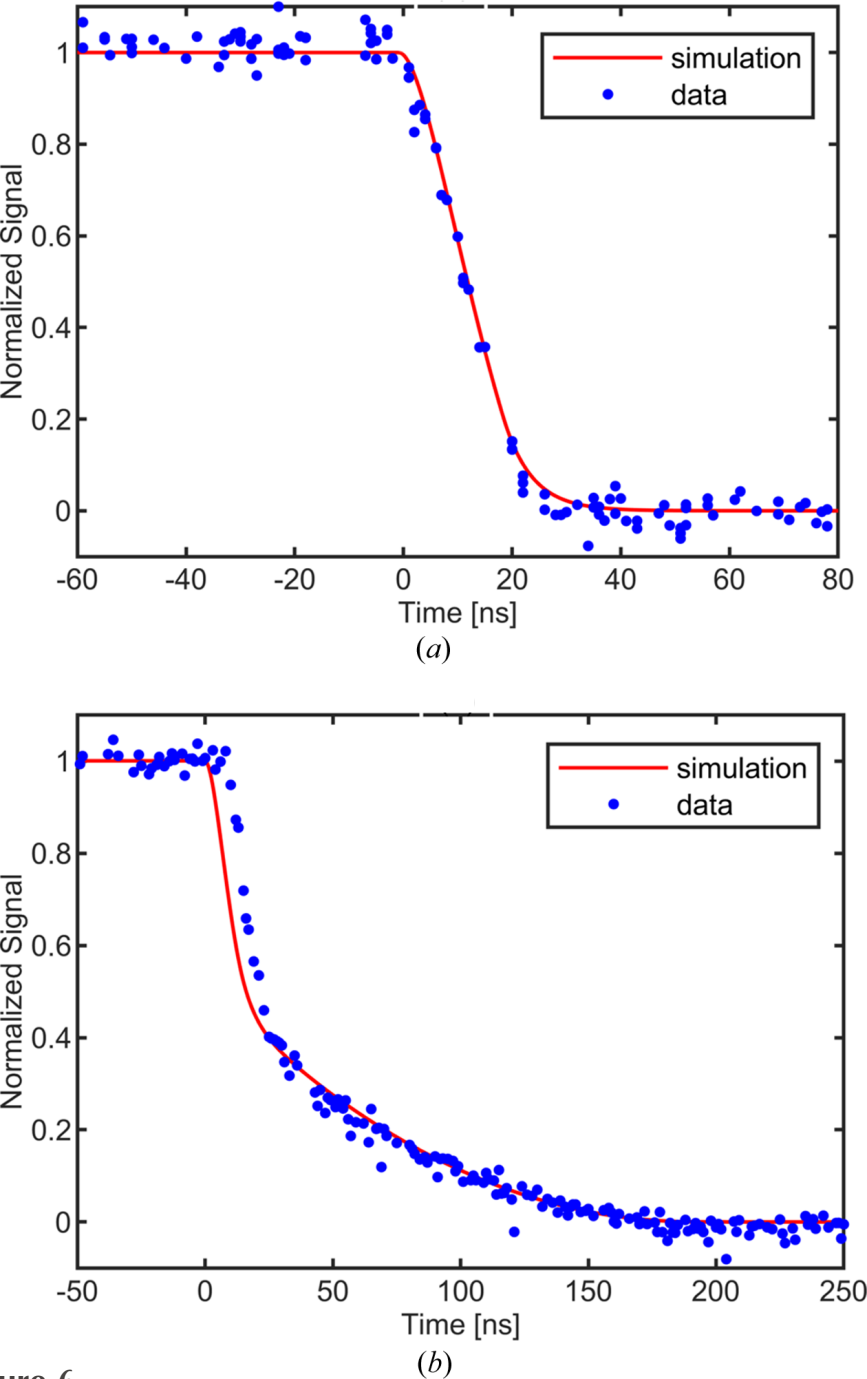
Integrated signal from an optical pulsed laser source as the phase delay slides the integration window past the overlap region with the induced current pulse. Mobilities extracted from data over a range of bias voltages are used to feed the simulation model to compare with the data. (*a*) The response to 515 nm illumination at −250 V bias. Energy is absorbed in the top of the sensor material, so the resulting current pulse is due entirely to electrons moving through the sensor. (*b*) The response to 1030 nm illumination at −300 V bias. Electron/hole pairs are produced uniformly throughout the sensor depth, yielding a response curve with components from both electrons and holes. The delay as the signal initially begins to fall is a consequence of the amplifier not keeping up at higher bias voltages. The duration of the falling edge of the pulse reflects the duration of the induced photocurrent inside the CdTe sensor.

Prior measurements of the mobility of electrons and holes in CdTe are typically 800–1200 cm^2^ V^−1^ s^−1^ and 70–110 cm^2^ V^−1^ s^−1^, respectively (Ariño-Estrada *et al.*, 2014 ▸). Even using the lowest measured mobilities, simulations suggest that over 90% of the induced current in electron-collecting CdTe should occur in 35 ns for X-ray energies up to 40 keV. To measure the electron and hole mobilities of our sensor material, we used a pulsed femtosecond optical laser at wavelengths of 1030 nm and 515 nm and scanned the integration window over the induced current pulse, as described previously. The laser spot had an area of approximately 4 mm^2^, and an area extending slightly past the spot was integrated to avoid any small pixel effects. The laser was operated at 1 kHz repetition rate, and a beamsplitter was used to illuminate both the detector and a fast photodiode. The fast photodiode plus a delay applied with a delay generator was used to trigger the detector to begin integration of the subsequent pulse, and the phase delay between the beginning of integration and the observed photon pulse was measured using a 300 MHz oscilloscope. The results are shown in Fig. 6 ▸. The attenuation length of 1030 nm photons in CdTe is large compared with the thickness of the sensor, meaning that electron–hole pairs are created nearly uniformly throughout the sensor layer. After electrons have drifted to their electrodes, a large tail due primarily to the movement of holes remains. Fitting this tail region to simulations yields a hole mobility of 100 ± 15 cm^2^ V^−1^ s^−1^ at 0°C. In contrast, at 515 nm, photons are absorbed in the top micrometre of the detector, so that the induced current is due almost exclusively to the movement of electrons. Comparing these results with simulations yields an electron mobility of 990 ± 100 cm^2^ V^−1^ s^−1^ at 0°C. Both of these values are consistent with past measurements of the properties of CdTe and suggest that 750 µm-thick CdTe will be an appropriate material for 77 ns imaging.

### 3.2. Single X-ray bunch characterization

**Figure 7:**
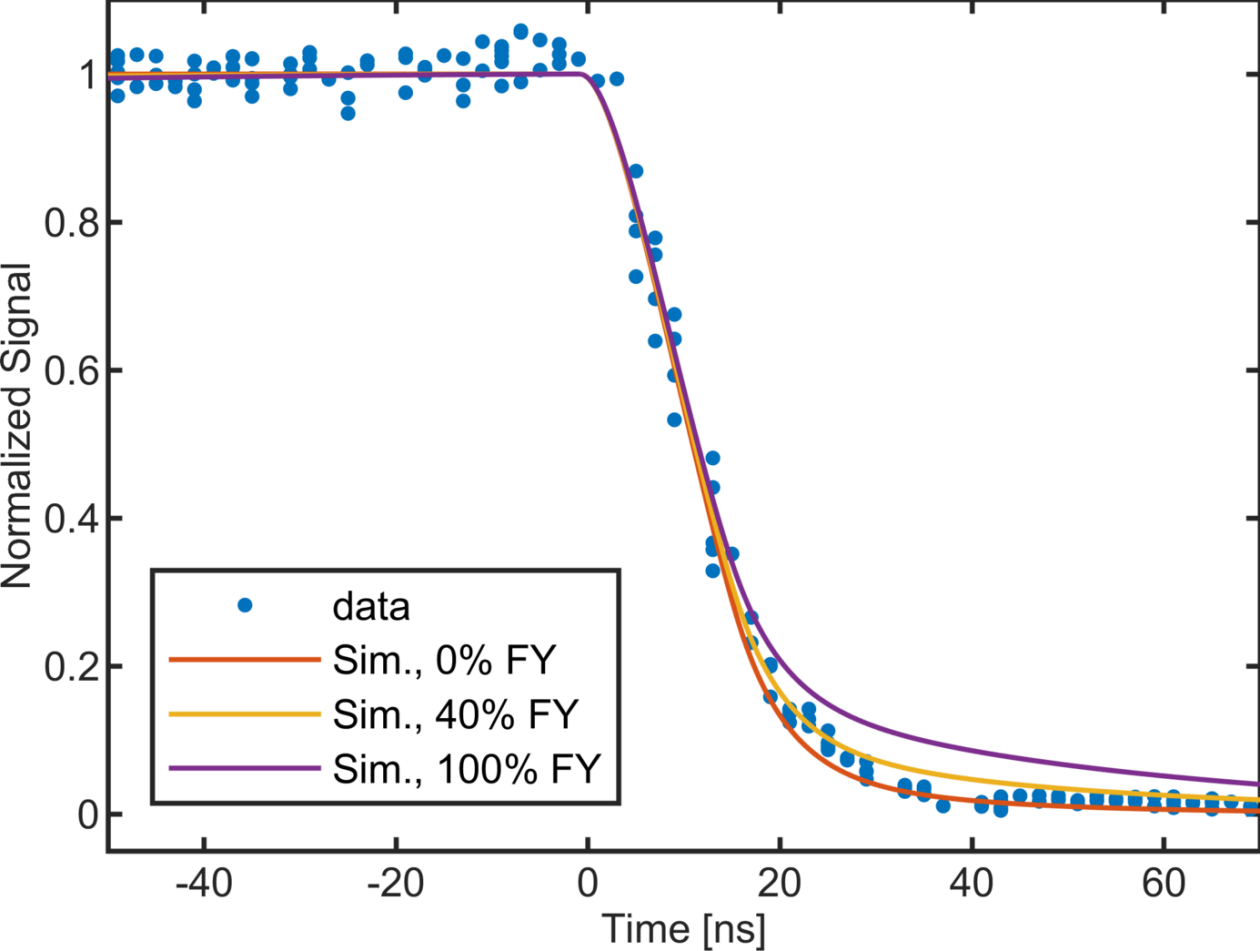
Observed and simulated normalized integrated signal plotted as a function of the phase delay between the photon pulse and the beginning of integration, for 29.2 keV photons. Simulations are performed assuming 0%, 40% and 100% of the absorbed photons produce a 23 keV fluorescent photon.

Data were also collected from single photon pulses produced at the FAST (Forming and Shaping Technology) Beamline ID3A at CHESS. The synchrotron was operated in nine-bunch mode with single bunches spaced approximately every 280 ns. A monochromator was used to isolate a primary energy of 29.2 keV. We sampled photons scattered by an aluminium scatterer from an unattenuated beam to understand the induced current for 29.2 keV photons. The region of interest on the detector had an area of around 30 mm^2^ and a mean signal level of 5 photons pixel^−1^ pulse^−1^. 29.2 keV is above the Cd *K*α edge at 26.7 keV, so fluorescence is expected in the sensor layer. The fluorescent photons are around 23 keV and thus have a longer attenuation length of around 120 µm compared with the primary photon’s attenuation length of 72 µm. These fluorescent photons can produce electron–hole pairs deeper in the sensor layer, which results in a prolonged induced current since these holes must travel a longer distance to their electrode. This results in a more pronounced tail in the induced current data and slower charge collection. The MATLAB simulation described previously was modified to produce the depth absorption profile and simulated induced current for a given fluorescent yield. Fluorescent photons travel through the sensor before being absorbed or escaping the sensor layer. Fig. 7 ▸ shows the measured intensity as a function of the phase between the X-ray bunch and the beginning of integration, compared with the results simulated assuming several different fluorescent yields. At a bias voltage of −400 V, 90% of the total induced current occurred within less than 35 ns. A hole-collecting sensor would require over 135 ns for this amount of charge collection at 400 V bias (Becker *et al.*, 2017 ▸).

**Figure 8:**
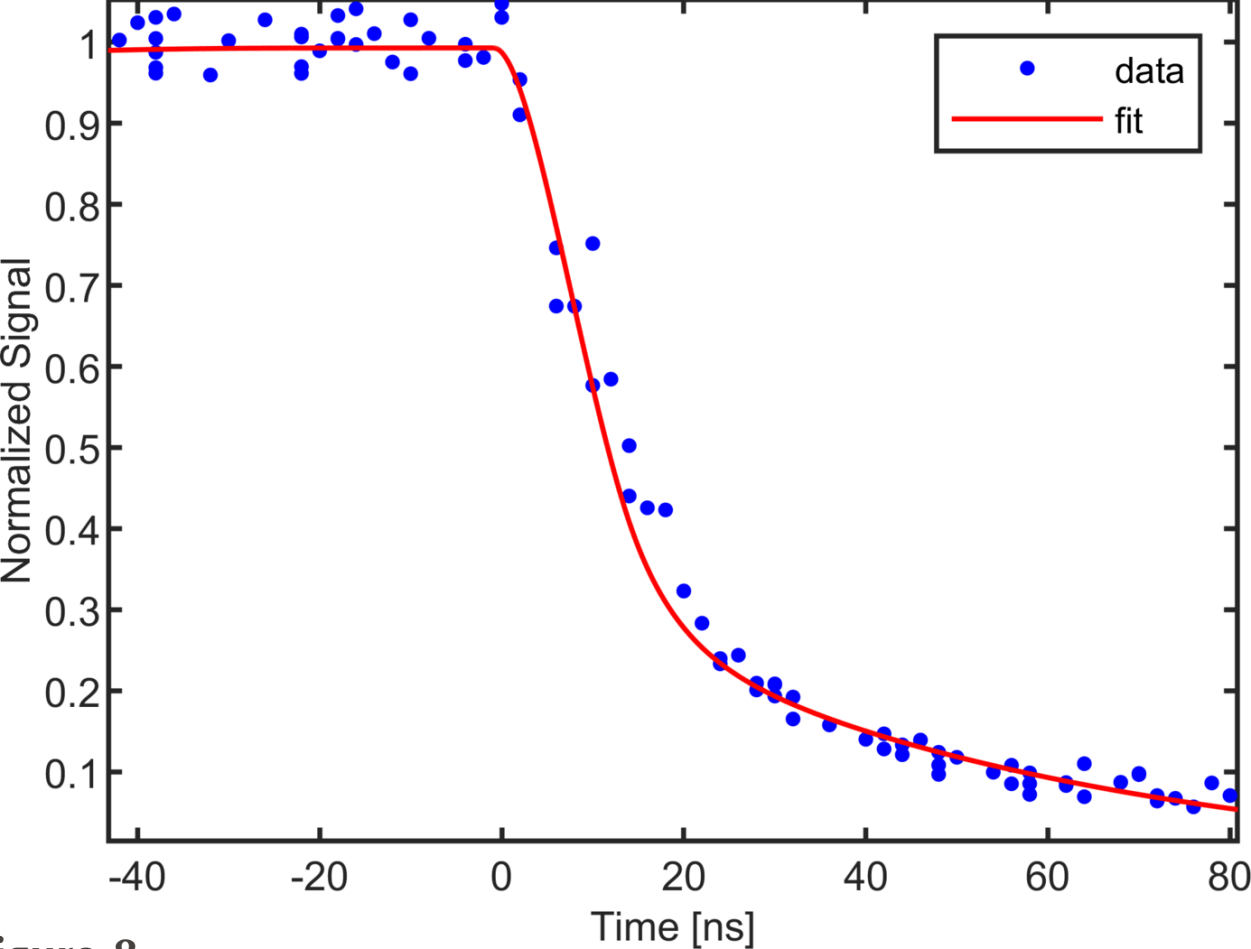
Observed and simulated normalized integrated signal plotted as a function of the phase delay between the X-ray pulse and the beginning of integration, for a highly attenuated 29.2 keV beam. At the attenuation used, much of the signal was from X-ray energies of 58.4 keV and higher. These photons convert at a much greater depth in the sensor, causing the current pulse from the holes to be extended over a greater period of time (∼80 ns for 95% charge collection).

We also studied a main beam attenuated by 5 mm of stainless steel. This attenuation eliminates 29.2 keV photons and leaves only higher-energy harmonics in the beam; the results are shown in Fig. 8 ▸. The attenuated beam had an area of 3 mm^2^, with a mean signal level of around 35 X-rays pixel^−1^ pulse^−1^. An area larger than the beam was integrated to avoid small pixel effects. The beam composition was measured from the spectra of scattered photons and found to consist of roughly 67% 58.4 keV photons, 20% 87.6 keV photons and 13% 116.8 keV photons. The response at these higher energies matches simulation well with 83% of the total signal integrated within the first 35 ns. Even higher-energy X-rays will be expected to produce even longer collection times, confirming the limited energy range for efficient charge collection at the higher frame rate. CdTe is frequently operated at bias voltages several hundred volts larger in magnitude than the −400 V used here for X-ray testing, and increased sensor bias would lead to proportionately faster charge collection. In this study, the bias voltage was limited to −400 V due to instability in the dark current at higher biases for this particular sensor sample.

## 4. Charge collection efficiency

**Figure 9:**
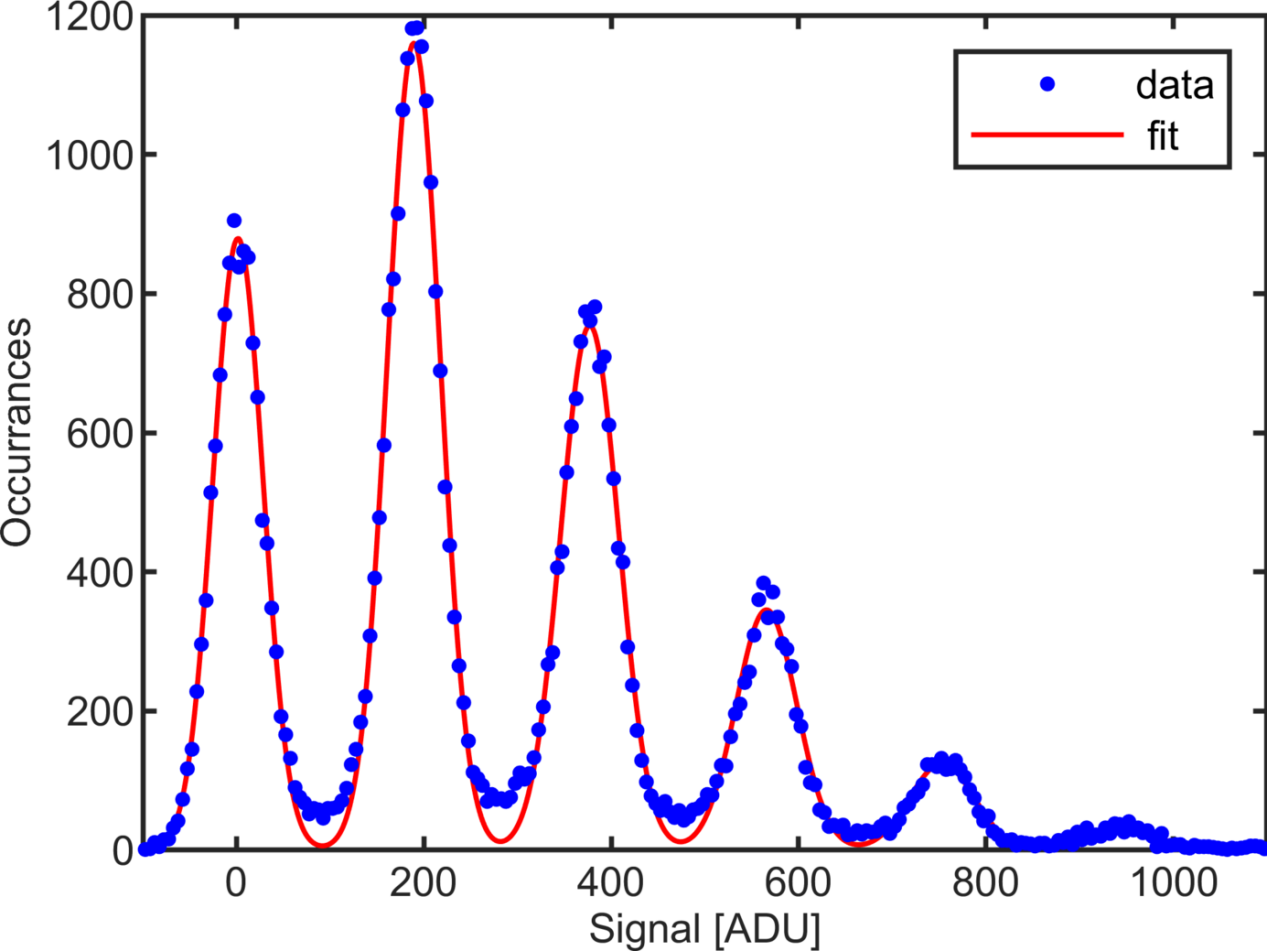
Photon spectra for 22.16 keV X-rays illuminating a single pixel through a pinhole mask. The first peak corresponds to 0 absorbed photons, the second to 1 absorbed photon *etc*. A sum of Gaussians constrained to have the same separation between maxima is fitted to determine a pixel gain of 8.5 ± 0.02 ADU keV^−1^ in high-gain mode for this sensor bonded to the CU-APS-PAD ASIC.

Charge collection efficiency was measured using the CU-APS-PAD in high-gain mode. An X-ray source with a silver anode illuminating a graphite monochromator was used to produce 22.16 keV photons. These were incident on a 150 µm-thick tungsten pinhole mask with 50 µm pinholes (smaller than the size of a single pixel) on a 0.44 mm pitch which was placed immediately in front of the detector. Analysis was performed on those X-ray spots that were contained within a single pixel. A histogram of background-subtracted pixel values is shown in Fig. 9 ▸. Each peak in the histogram represents a pixel which has absorbed a discrete number of photons (for example, the peak centered at 0 corresponds to 0 photons, the first peak to the right, 1 photon, up to 5 photons in Fig. 9 ▸). The peak spacing gives a conversion factor of 8.5 ± 0.02 ADU keV^−1^ for this CdTe. Previous measurements with this system have measured 11.2 ± 0.1 ADU keV^−1^ for a silicon sensor and 8.32 ADU keV^−1^ for hole-collecting CdTe (Gadkari *et al.*, 2022 ▸). The ratio of CdTe gain to Si gain should be equal to the ratio between the electron–hole pair creation energies of Si to CdTe, 0.817 (Kolanoski & Wermes, 2022 ▸). While the electron- and hole-collecting sensors produced comparable results, the ratio between the CdTe and Si is lower than expected, only 0.76. This could either be due to the presence of trapping within the CdTe or to a higher input capacitance of the CdTe sensor relative to the silicon sensor, resulting in a lower fraction of charge being collected on the input stage.

## 5. CdTe sensor polarization

**Figure 10:**
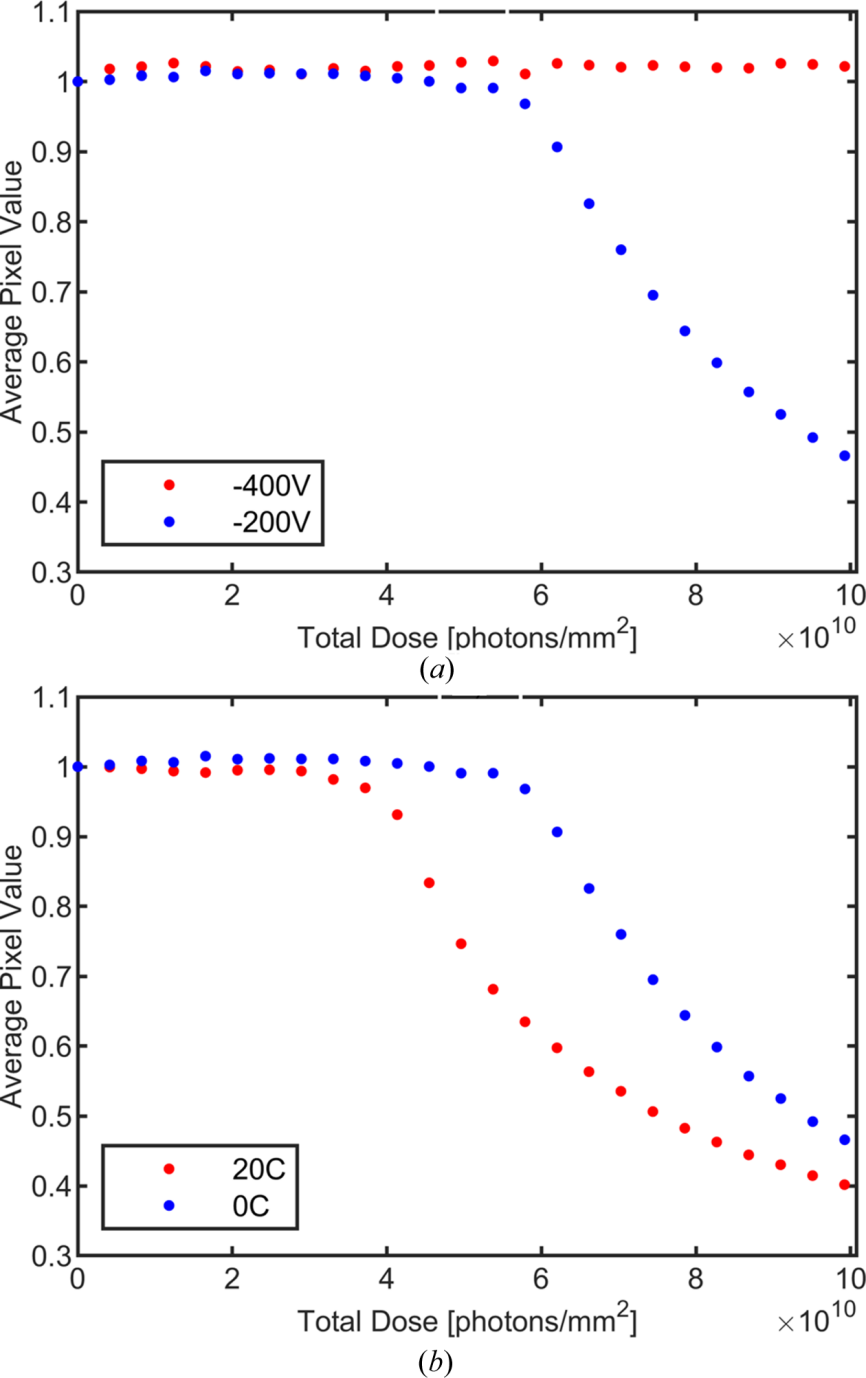
Mean pixel values recorded on a sensor under flood-illumination with 2 × 10^7^ photons mm^−2^ s^−1^, normalized by the mean pixel value of an unpolarized sensor. In (*a*) the sensor temperature is maintained at 0°C and the bias voltage is varied. In (*b*) the sensor bias is maintained at −200 V and the sensor temperature is varied.

The performance of CdTe sensor material is degraded by polarization effects. Polarization can be induced both by radiation and by biasing the sensor. In the case of radiation-induced polarization, charge carriers generated by the absorbed radiation become trapped, and space charge builds up in the sensor layer. In bias-induced polarization, electrons from the cathode accumulate near the positive contact (Owens, 2019 ▸). Both forms of polarization disrupt the applied electric field and decrease charge collection, which in charge-integrating detectors manifests as a decrease in measured signal at a given X-ray intensity. To study the effects of polarization we performed flood-illumination of the sensor using a silver anode X-ray tube located around 5 cm from the sensor and took images as dose was accumulated by the sensor. The photon flux on the detector was 2.5 × 10^7^ 20 keV photons mm^−2^ s^−1^ and the sensors were illuminated for around 1.5 h. Fig. 10 ▸(*a*) shows the normalized pixel signal for a sensor held at 0°C and biased at −200 V or −400 V. Fig. 10 ▸(*b*) shows the normalized pixel signal as a function of the dose per mm^2^ for a sensor biased at −200 V and held at either 0 or 20°C. Twenty-four images with 100 µs integration time were taken and averaged at each time interval. Polarization, indicated by a decrease in normalized average signal, occurs more quickly at −200 V bias and at higher detector operating temperatures. Slower polarization at higher biases and lower temperatures was also observed in hole-collecting CdTe in past measurements conducted by the group (Becker *et al.*, 2016 ▸).

**Figure 11:**
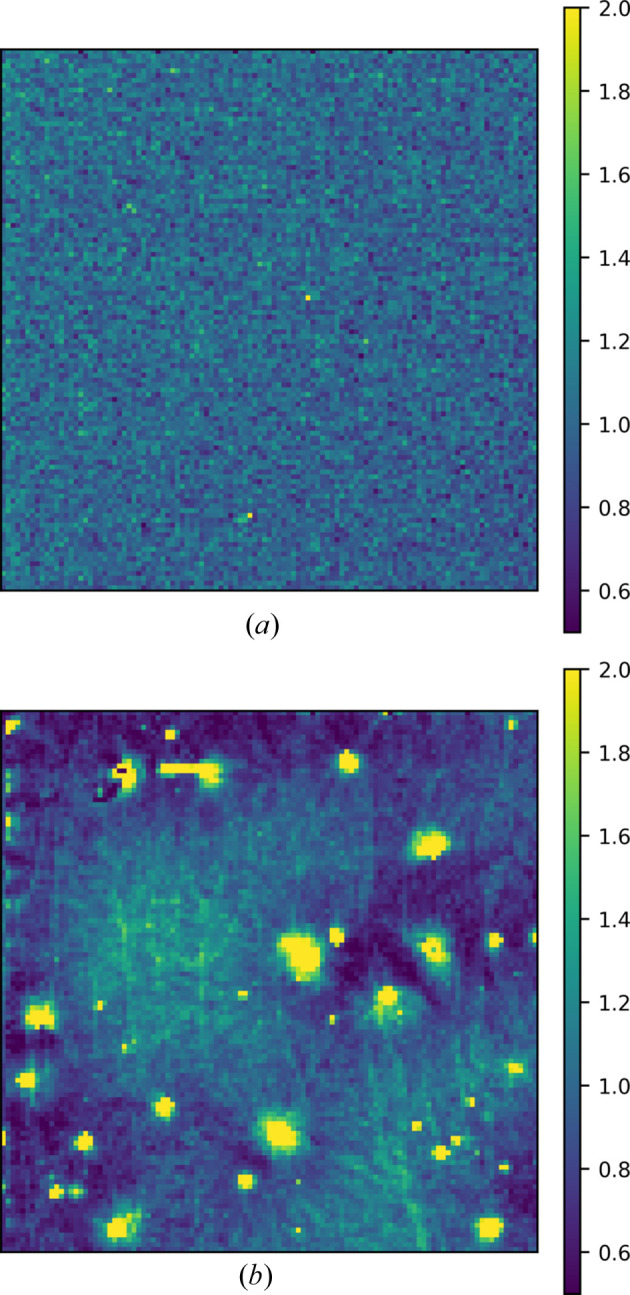
Images taken under flood-illumination before (*a*) and after (*b*) 1.5 h of constant flood-illumination at −200 V bias at 0°C. Pixel values are normalized to the mean in each image. The total dose accumulated in the sensor between the two images was 1.3 × 10^11^ 20 keV photons mm^−2^.

Polarization can also degrade the uniformity of response. Fig. 11 ▸ shows images normalized by their mean pixel values which are taken before and after illumination with 10^11^ 20 keV photons mm^−2^ (1.5 h of illumination) at −200 V bias. The images were obtained by flood-illuminating the sensor with a silver anode X-ray tube at a distance of around 5 cm. The image taken after dosing [Fig. 11 ▸(*b*)] shows a network of lines as well as spots with high signal. Past work on hole-collecting CdTe showed a line network after polarization, but no high-signal spots (Becker *et al.*, 2016 ▸). Since CdTe exhibits both radiation-induced and bias-induced polarization, the changes are likely a combination of both phenomena. After exposure to a dose of 10^11^ photons mm^−2^, the mean pixel value decreased by 58% and the standard deviation of the pixels increased from 16% of the mean to 85% of the mean. We note that the reported variation in the sensor after polarization is a lower bound since certain bright regions of the image are saturated.

## 6. Conclusions

To produce a detector capable of imaging from sequential 40 keV synchrotron X-ray bunches separated by 77 ns, collection of the charge should occur within 35 ns, reserving the remainder of the 77 ns imaging period for other processes in the detector electronics. As such, we want a minimum of 90% of the induced current to be collected within the 35 ns integration period.

High-quality CdTe suitable for use in pixel array detectors is currently commercially available in a minimum thickness of 750 µm. At this thickness, the quantum efficiency of the material at 40 keV is near 100%, with over 90% efficiency maintained up until around 65 keV. Hole-collecting CdTe in this thickness has been used to perform time-resolved experiments with sub-microsecond resolution (Becker *et al.*, 2017 ▸). However, the speed of 750 µm hole-collecting CdTe is limited by the mobility of holes in the material, especially for X-rays with attenuation lengths much shorter than the sensor thickness. When X-rays with attenuation lengths short relative to the sensor thickness (*e.g* 40 keV at around 90 µm) are absorbed, the more rapid induced current pulse in electron-collecting CdTe allows for single-bunch imaging at higher bunch frequency. Electron-collecting 750 µm CdTe will allow for imaging of bunches separated by 77 ns which will occur at the upgraded Advanced Photon Source.

Investigations into the polarization of electron-collecting CdTe sensor material suggest that at fluxes around 10^7^ photons mm^−2^ s^−1^, both electron- and hole-collecting CdTe polarize at similar rates as indicated by the decay of the mean pixel value at constant illumination, with higher bias voltages and lower temperatures serving to delay the onset of polarization. Images taken before and after polarization using electron-collecting CdTe show a network of lines which are also observed in hole-collecting CdTe; however, electron-collecting CdTe exhibits additional spots with high measured signal. More work is needed to understand the reason for these spots and the difference between the two materials.

## References

[bb55] Ariño-Estrada, G., Chmeissani, M., de Lorenzo, G., Kolstein, M., Puigdengoles, C., García, J. & Cabruja, E. (2014). *J. Instrum.* **9**, C12032. 10.1088/1748-0221/9/12/C12032 PMC4340550 25729405

[bb1] Becker, J. (2010). PhD thesis, University of Hamburg, Germany.

[bb2] Becker, J., Tate, M., Shanks, K., Philipp, H., Weiss, J., Purohit, P., Chamberlain, D. & Gruner, S. (2017). *J. Instrum.* **12**, P06022.

[bb3] Becker, J., Tate, M., Shanks, K., Philipp, H., Weiss, J., Purohit, P., Chamberlain, D., Ruff, J. & Gruner, S. (2016). *J. Instrum.* **11**, P12013.

[bb4] Gadkari, D., Shanks, K., Hu, H., Philipp, H., Tate, M., Thom-Levy, J. & Gruner, S. (2022). *J. Instrum.* **17**, P03003.

[bb5] Kolanoski, H. & Wermes, N. (2022). *Particle Detectors: Fundamentals and Applications.* Oxford University Press.

[bb6] Lambert, P. K., Hustedt, C. J., Vecchio, K. S., Huskins, E. L., Casem, D. T., Gruner, S. M., Tate, M. W., Philipp, H. T., Woll, A. R., Purohit, P., Weiss, J. T., Kannan, V., Ramesh, K. T., Kenesei, P., Okasinski, J. S., Almer, J., Zhao, M., Ananiadis, A. G. & Hufnagel, T. C. (2014). *Rev. Sci. Instrum.* **85**, 093901. 10.1063/1.4893881 PMC4156581 25273733

[bb7] Owens, A. (2019). *Semiconductor Radiation Detectors.* CRC Press.

[bb8] Philipp, H. T., Tate, M. W., Purohit, P., Chamberlain, D., Shanks, K. S., Weiss, J. T. & Gruner, S. M. (2016*a*). *AIP Conf. Proc.* **1741**, 040036.

[bb9] Philipp, H. T., Tate, M. W., Purohit, P., Shanks, K. S., Weiss, J. T. & Gruner, S. M. (2016*b*). *J. Synchrotron Rad.* **23**, 395–403. 10.1107/S1600577515022754 PMC4768764 26917125

